# Impact of the COVID-19 pandemic on emergency department attendances and acute medical admissions

**DOI:** 10.1186/s12873-021-00529-w

**Published:** 2021-11-20

**Authors:** Michael E. Reschen, Jordan Bowen, Alex Novak, Matthew Giles, Sudhir Singh, Daniel Lasserson, Christopher A. O’Callaghan

**Affiliations:** 1 Department of Acute General Medicine, John Radcliffe Hospital, Oxford University Hospitals NHS Foundation Trust, Headley Way, OX3 9DU Oxford, UK; 2 Emergency Medicine Research Oxford (EMROx), John Radcliffe Hospital, Oxford University Hospitals NHS Foundation Trust, Headley Way, Oxford, OX3 9DU UK; 3 Nuffield Department of Medicine, NIHR Oxford Biomedical Research Centre, Old Road Campus, Oxford, OX3 7BN UK

**Keywords:** COVID-19, Emergency department, Acute medicine, Hospital admissions, Non-COVID-19 disease

## Abstract

**Background:**

To better understand the impact of the COVID-19 pandemic on hospital healthcare, we studied activity in the emergency department (ED) and acute medicine department of a major UK hospital.

**Methods:**

Electronic patient records for all adult patients attending ED (*n* = 243,667) or acute medicine (*n* = 82,899) during the pandemic (2020–2021) and prior year (2019) were analysed and compared. We studied parameters including severity, primary diagnoses, co-morbidity, admission rate, length of stay, bed occupancy, and mortality, with a focus on non-COVID-19 diseases.

**Results:**

During the first wave of the pandemic, daily ED attendance fell by 37%, medical admissions by 30% and medical bed occupancy by 27%, but all returned to normal within a year. ED attendances and medical admissions fell across all age ranges; the greatest reductions were seen for younger adults in ED attendances, but in older adults for medical admissions. Compared to non-COVID-19 pandemic admissions, COVID-19 admissions were enriched for minority ethnic groups, for dementia, obesity and diabetes, but had lower rates of malignancy. Compared to the pre-pandemic period, non-COVID-19 pandemic admissions had more hypertension, cerebrovascular disease, liver disease, and obesity. There were fewer low severity ED attendances during the pandemic and fewer medical admissions across all severity categories. There were fewer ED attendances with common non-respiratory illnesses including cardiac diagnoses, but no change in cardiac arrests. COVID-19 was the commonest diagnosis amongst medical admissions during the first wave and there were fewer diagnoses of pneumonia, myocardial infarction, heart failure, cellulitis, chronic obstructive pulmonary disease, urinary tract infection and other sepsis, but not stroke. Levels had rebounded by a year later with a trend to higher levels of stroke than before the pandemic. During the pandemic first wave, 7-day mortality was increased for ED attendances, but not for non-COVID-19 medical admissions.

**Conclusions:**

Reduced ED attendances in the first wave of the pandemic suggest opportunities for reducing low severity presentations to ED in the future, but also raise the possibility of harm from delayed or missed care. Reassuringly, recent rises in attendance and admissions indicate that any deterrent effect of the pandemic on attendance is diminishing.

**Supplementary Information:**

The online version contains supplementary material available at 10.1186/s12873-021-00529-w.

## Background

In 2020 Severe Acute Respiratory Syndrome Coronavirus 2 (SARS-CoV-2) caused a worldwide pandemic of COVID-19 disease resulting in substantial excess mortality and global disruption to healthcare and social care. The first major peak in COVID-19 cases in England occurred between March and May in 2020 and prompted a national lockdown. Lockdown measures were adjusted during the pandemic and widespread vaccination in 2021 triggered relaxation of the restrictions. During the pandemic, healthcare was rapidly restructured in anticipation of predicted needs [1]. This included redeployment of people and resources, especially to acute general medicine, emergency medicine and critical care, reductions in non-COVID-19 research activity, reductions in elective procedures, and increased use of remote telephone or video consultations [2, 3].

Reports of reductions in hospitalization for non-COVID-19 acute illnesses have raised concerns that patients may not have attended hospital for an acute illness and might subsequently experience increased morbidity or mortality as a result [4–9]. Factors influencing hospital attendance during the pandemic may have included fear of acquiring COVID-19 infection, a desire to reduce the pressure on hospitals or a higher threshold among referring and receiving clinicians for hospital review or admission. Conversely, others have suggested patients avoiding ED had more minor illness and this had a beneficial effect by reducing crowding in ED [10].

In the UK, acute hospital care is provided by direct presentation of patients to the emergency department (ED) or referral of patients by their primary care general practitioner or paramedics to the hospital [11]. We sought to understand both the COVID-19 and non-COVID-19 activity in the ED and the acute medicine department and how this changed across the course of the pandemic. It is important to understand both the dramatic changes that occurred during the first wave of the pandemic and the subsequent patterns of acute care usage after this early phase.

The aim of our study was to determine the clinical characteristics of emergency department attendances and medical admissions during the COVID-19 pandemic and whether particular groups were under-represented during the pandemic peaks and the time frame of any changes.

We identify and characterise major changes in ED attendances and in medical admissions during the pandemic and highlight changes in physiological severity, patterns of diagnoses, and outcomes including mortality. These findings demonstrate the profound impact of a pandemic on urgent care, even for non-pandemic illness, and will form a foundation for planning to minimise the impact in the future.

## Methods

We extracted data from the hospital electronic health record (EHR) for all ED patients aged 18 or over from the Oxford University Hospitals NHS Foundation Trust (John Radcliffe and Horton sites) between 17th March 2019 and 12th July 2021 (*n* = 243,667). We extracted data for medical admissions from the EHR between 1st January 2019 and 18th August 2021 and pruned the analysis to 17th March to 18th July 2021 (*n* = 82,899). The longer time period for admissions compared to ED attendances allows proper analysis of medical patients who were inpatients during the period of interest but admitted or discharged outside this time period. ED attendances and acute medicine patient data incorporate critical care and high dependency unt (HDU) admissions because these admissions pass through ED or for medical patients remain under the duty medical physician. Day-case attendances (consisting of procedural admissions for e.g., endoscopy or bronchoscopy) were removed from the data by filtering out admissions to a day-case unit location or where the admission method was ‘planned’, ‘booked’, or ‘elective’. For analysis of acute medical patients, the hospital attendances were sub-divided into ‘medical attendances’ where the patient was discharged directly from the ambulatory emergency care unit (AEC) and into ‘medical admissions’ where the patient was admitted directly to a bed-based pathway or admitted to a bed-based pathway from the AEC. At our institution medical admissions mostly occur through the emergency medical assessment unit (EAU) but can also occur directly from ED to medical wards or directly from the ambulance service to cardiology for suspected ST-elevation myocardial infarction.

Clinical ED data are recorded according to the UK emergency care dataset (ECDS) parameters [12]. The ED diagnosis is recorded in real time by the clinician selecting from a curated list of SNOMED terms. We modified this list by adding diagnostic codes for COVID-19 and categorizing COVID-19 into the ‘respiratory’ group of diseases in group 2 of the ECDS diagnostic tree (Supplementary file ECDS data – table of ECDS codes and groupings).

For acute medical admissions the diagnosis of COVID-19 was derived from the primary diagnosis data field using ICD-10 codes of U07.1 (COVID-19, virus identified) and U07.2 (COVID-19, virus not-identified). The medical diagnoses are recorded by professional medical coders after the admission is completed using aggregated data from the EHR. Inpatient COVID-19 diagnoses in our institution are generally made by a consultant using a combination of clinical data, PCR testing, lateral flow testing and chest X-ray or CT findings. For ED patients, PCR testing was not widely available during the first wave of the pandemic.

Mortality data were obtained from the EHR and by querying the NHS Digital Personal Demographics Service Database using the Demographics Batch Service [13].

Our hospitals serve a population of around 650,000. The UK containment phase of the pandemic ended on 12th March 2020 and from 16th March onwards a ‘lockdown’ was officially advised and enforced from 23rd March 2020. We defined the first wave of the pandemic period as from 17th March 2020 to 31st May 2020 and this captures the major first peak of COVID-19 in the UK. Patients with suspected COVID-19 (fever, respiratory symptoms) or confirmed COVID-19 are assigned into side-rooms, grouped bays, or designated wards according to a traffic light system of Green (not COVID-19), Amber (suspected COVID-19) or Blue (confirmed COVID-19). Further details on COVID-19 infection control and pathways at our Institution are published elsewhere [14]. We define the second wave of the pandemic period as from 26th November 2020 to 10th February 2021. For numerical comparisons we used a pre-pandemic period in 2019 that matched the first pandemic peak and a late-pandemic period matching the equivalent time period in 2021 one calendar year after the first peak period.

Analysis was undertaken using R [15]. Rolling averages over time were calculated using the ‘rollmean’ function of the zoo (v1.8.9) package in R [16]. A centred rolling window of 14 days was used for daily deaths and daily medical admissions and a window of 28 days for all other plots. ED patients were considered to be ‘Admitted’ if admitted from ED for more than 24 h or ‘Discharged’ if discharged from ED directly or within 24 h of attending hospital. For medical attendances diagnoses were stratified as COVID-19 if the primary diagnosis code was either U07.1 or U07.2 and as non-COVID-19 for all other primary diagnoses.

For age group analysis patients were stratified into 10 age groups of equal time width using the binning function in the R package dlookr [17]. For categorical variables including gender and ethnicity, the difference in distribution between the pre-pandemic period and pandemic first wave peak period was compared with a chi-squared test and if significant, then a row-wise proportion test with multiple testing correction to evaluate the difference between categories using the prop test function in the rstatix package [18]. To calculate distance from a patient’s domiciliary address to hospital we used the code for the UK census area of their address, termed the ‘lower layer super output area’ (LSOA) and obtained the latitude and longitude for the centroid position of the LSOA from the 2011 Office of National Statistics Census data [19]. To obtain the latitude and longitude from the Northing and Easting positions in the census data we converted them using the web tool https://gridreferencefinder.com/batchConvert/batchConvert.php. To calculate the straight-line distance from the hospital to the respective centroid coordinate we used a webtool distance calculator: https://stevemorse.org/nearest/distancebatch.html. The significance comparison of distance was calculated with a Student’s t test. A deprivation decile was assigned by matching the LSOA code to the Index of Multiple Deprivation data from the English Indices of Deprivation 2015 [20]. A value of 1 indicates the most deprived area and an overall chi squared test was performed between comparator groups and if significant then a row-wise proportion test.

For medical patients, co-morbidities were determined from the ICD10 codes for secondary diagnoses up to a depth of 50 co-morbidities. A combined co-morbidity score was calculated according to the mean weighted Elixhauser score system using the R package ‘comorbidity’ [21, 22]. We amalgamated the comorbidity R package categories of ‘hypertension’ and ‘hypertension-uncomplicated’ as well as ‘diabetes’ and ‘diabetes-complicated’. Alcoholic liver disease was parsed separately using an ICD10 code of K70. To calculate a NEWS2 score (National Early Warning Score 2) we used the first set of observations including temperature, heart rate, systolic/diastolic blood pressure, peripheral oxygen saturation (without correction for chronic type 2 respiratory failure status), Glasgow Coma Score and the use of supplemental oxygen [23]. We report mean scores compared using a Wilcoxon Rank sum test, and calculated incidence rate ratios for each of the 4 NEWS2 alert categories. NEWS2 score were binned into the NEWS2 clinical risk alert levels whereby a score of 0–4 = “low”, unless any individual category is 3 in which case = “low-medium”, 5–6 = “medium”, 7+ = “high”. The number of attendances/admissions without available observations is shown in supplementary tables referred to in the Results. To calculate an incidence rate ratio between the pandemic and pre-pandemic period we used a Poisson regression model of daily counts of each category implemented by the R mfx package ‘poisonirr’ [7, 24]. The proportion of patients using oxygen was compared with a chi-squared test.

For the ED presenting complaint and primary diagnosis we selected the 10 commonest diagnoses across the whole dataset. The ED primary diagnosis data is displayed at the 2nd group level of the ECDS system (with COVID-19 grouped in ‘Respiratory’). For the primary diagnosis for medical admissions, we selected the 10 commonest primary diagnoses at the ICD-10 tier level of 3 alphanumeric characters. Statistical analysis was performed using incidence rate ratios as described above. For ED data we also show a table of manually selected diagnoses at the most granular level of diagnosis. Student’s t-test was used to analyse ED length of stay. For ED mortality we calculated a mortality rate using the number of patients who died during their ED attendance or before leaving hospital if they were admitted and applied a chi-squared test of the proportion. For medical patients we calculated the mortality rate during admission and compared the proportion of admissions with mortality using a chi-squared test.

The study formed part of an institutional service evaluation using retrospectively collected anonymized routine clinical data and was deemed not to require further ethical approval or informed consent from patients.

## Results

### Impact on ED attendances and acute medical admissions

**Fig. 1:**
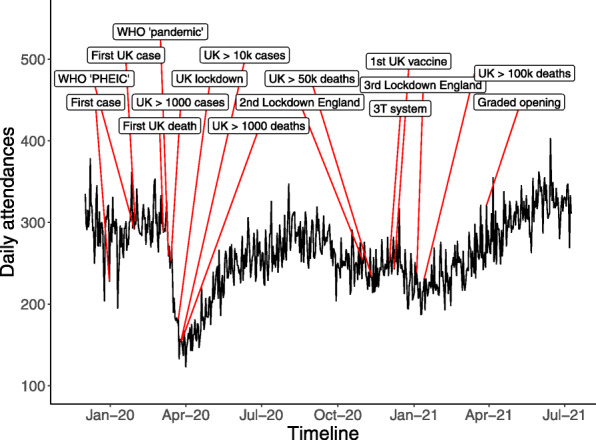
Daily ED attendance over time with major event time points labelled (WHO=World Health Organization, PHEIC=Public health emergency of international concern, 3 T system = 3-tiered regional lockdown system)

ED attendances declined sharply following the first UK death from COVID-19, the reporting of over 1000 UK cases, and the World Health Organisation (WHO) declaring a pandemic (Fig. 1). During the first wave of the pandemic, there was a 37% fall in total ED attendances to 195 per day from 309 per day in the matched period of the preceding year and this rose to 296 per day in the matched period of 2021.

**Fig. 2:**
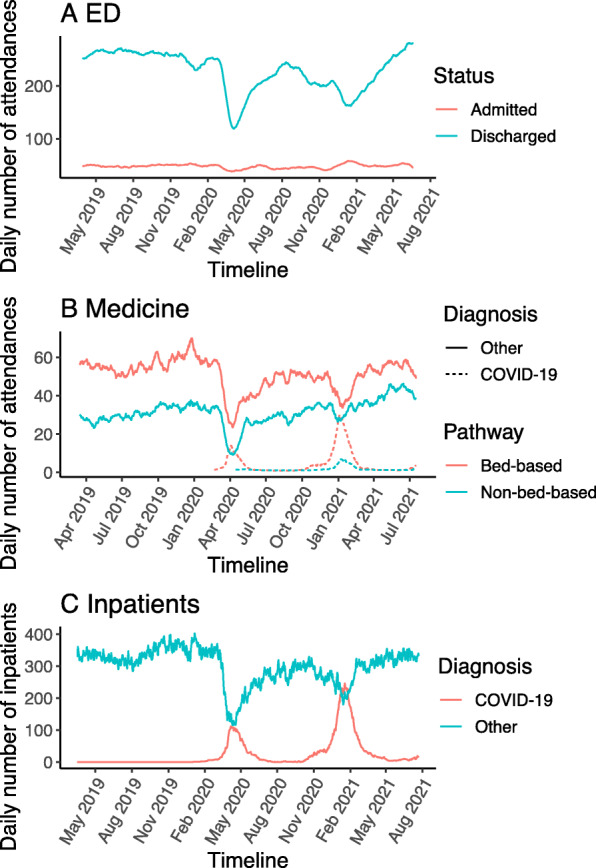
Impact of the pandemic on ED attendances and medical admissions. (**A**) Attendances to ED stratified by discharge home within 24 h versus admission for > 24 h. (**B**) Attendances (non-bed based) and admissions (bed-based) stratified by primary diagnosis of COVID-19. (**C**) Daily number of medical inpatients stratified by primary diagnosis of COVID-19

The daily number of hospital admissions arising from all ED attendances was 50 in the matched period in 2019, falling to 42 during the first wave pandemic and rising again to 49 in the matched period in 2021 (Fig. 2A). The timeline shows attendances that did not lead to admission dipped during the periods of national peaks in COVID-19 cases, but then rose to the highest levels during the summer of 2021 after most lockdown measures had ended (Fig. 2A).

Daily attendances to the acute medical department fell by 30% from 83 in the matched period in 2019 to 58 during the first wave and rose to 96 in the matched period in 2021 (Fig. 2B). Acute medical admissions per day also fell by 28% from 56 in the matched period of 2019 to 40 in the first wave, rising to 56 in the matched period in 2021. We further selectively analysed medical patients who were admitted into a bed-based pathway because planning inpatient bed use during the pandemic was a key challenge to ensure that the hospital was not overwhelmed. The first peak in COVID-19 admissions occurred in the week commencing 29th March 2020, when there were 113 (245 non-COVID-19 admissions) and the second peak occurred in the week commencing 3rd January 2021 with 266 (455 non-COVID-19 admission) (Fig. 2B).

The number of medical inpatients in the hospital each day fell from a mean of 334 in the pre-pandemic period to 244 in the first peak, 376 in the second peak and 329 in 2021 (Fig. 2C). Overall, the percentage of medical beds occupied by patients with COVID-19 rose to 26.5% during the first peak and 36.2% in the second peak.

### Clinical characteristics of ED attendances and medical admissions

**Fig. 3:**
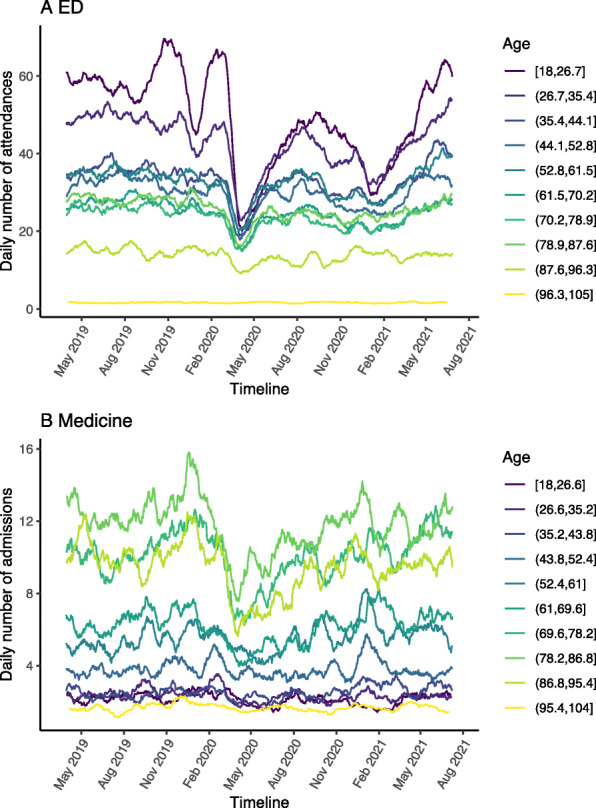
ED attendances (**A**) and medical admissions (**B**) stratified into 10 bands of equal age-width (square brackets indicate inclusive, round brackets indicate not inclusive)

A significant reduction in total ED attendances was observed across all age groups in the first wave of the pandemic with the biggest absolute and relative reductions among the two youngest age bands (Fig. 3A, Table 1, Supplementary Table 1). The timeline shows an increase in attendances for the younger age groups towards pre-pandemic levels by the summer of 2020. The mean age increased significantly from 50 in the pre-pandemic period to 53 in the pandemic first wave (Supplementary Table 1). There was no change in the distribution of gender or deprivation indices during the pandemic period first wave compared to the matched pre-pandemic period in 2019 (Supplementary Table 1). The mean distance from the patient’s home to the hospital for ED attendances fell from 19.2 km in the pre-pandemic period to 16.1 km in the pandemic period (*p* < 0.001). For ethnicity there was an increase in the proportion with ethnicity ‘Mixed – White and Asian’, ‘White – British’, and decrease in ‘White – Any other White Background’ (Supplementary Table 1).

**Table 1:** Number of ED attendances by age band during the pandemic first wave period and the same time period in 2019

Age band	Pre-pandemic (%^1^)	1st pandemic wave (%^1^)	Difference	Incidence rate ratio	Confidence interval	*P* value
18–26.7	4519 (19.2)	1951 (13.2)	2568	0.43	0.38–0.49	< 0.001
> 26.7 ≤ 35.4	3727 (15.9)	2129 (14.4)	1598	0.57	0.52–0.62	< 0.001
>35.4 ≤ 44.1	2685 (11.4)	1794 (12.1)	891	0.67	0.59–0.75	< 0.001
>44.1 ≤ 52.8	2422(10.3)	1740 (11.7)	682	0.72	0.63–0.80	< 0.001
>52.8 ≤ 61.5	2650 (11.3)	1933 (13.0)	717	0.73	0.64–0.82	< 0.001
>61.5 ≤ 70.2	2051 (8.7)	1448 (9.7)	603	0.71	0.62–0.79	< 0.001
>70.2 ≤ 78.9	1984 (8.4)	1401 (9.4)	583	0.71	0.61–0.80	< 0.001
>78.9 ≤ 87.6	2201 (9.4)	1547 (10.4)	654	0.70	0.61–0.79	< 0.001
>87.6 ≤ 96.3	1167 (5.0)	827 (5.6)	340	0.71	0.62–0.80	< 0.001
>96.3 ≤ 105	94 (0.4)	58 (0.4)	36	0.62	0.39–0.85	0.011

^1^Percentage of attendances in age band compared to total for all age bands

In contrast to ED attendances, most medical admissions in the pre-pandemic period were in the older age groups (Fig. 3B, Table 2, Supplementary Table 2). During the first wave pandemic period the absolute numbers of medical admissions fell across all ages (Table 2). The mean age was significantly lower at 69 in the pandemic period (non-COVID-19) compared to 71 in the pre-pandemic period (Supplementary Table 2). There was no difference in mean age within the pandemic first wave between non-COVID-19 (69) and COVID-19 admissions (69). There was no significant difference in the gender or deprivation profiles between the pre-pandemic period and the pandemic first wave period (Supplementary Table 2). Among non-COVID-19 admissions during the pandemic 1st wave peak there was in increase in the proportion with ethnicity ‘Other -Not stated’. Compared with non-COVID-19 patients, admissions for patients with COVID-19 had a higher proportion of ethnicity ‘Asian - Any Other Asian Background’, ‘Black or Black British - African’, ‘Other - Not Stated’ and a reduction in ‘White - British’ (Supplementary Table 2). The mean distance from the patient’s home to the hospital for all medical admissions (including COVID-19) did not differ significantly from 17.6 km in the pre-pandemic period to 16.4 km during the pandemic period.

**Table 2:** Number of medical admissions by age in patients without COVID-19 before and during the pandemic first wave period

Age band	Pre-pandemic (%^1^)	1st pandemic wave (%^1^)	Difference	Incidence rate ratio	Confidence interval	*P* value
18–26.6	136 (3.2)	84 (3.3)	52	0.62	0.39–0.84	0.009
>26.6 ≤ 35.2	154 (3.6)	98 (3.8)	56	0.64	0.40–0.87	0.015
>35.2 ≤ 43.8	176 (4.1)	120 (4.7)	56	0.68	0.43–0.94	0.042
>43.8 ≤ 52.4	263 (6.2)	207 (8.1)	56	0.79	0.64–0.93	0.01
>52.4 ≤ 61	383 (9.0)	260 (10.2)	123	0.68	0.58–0.77	< 0.001
>61 ≤ 69.6	477 (11.2)	291 (11.4)	186	0.61	0.50–0.72	< 0.001
>69.6 ≤ 78.2	788 (18.6)	460 (18.0)	328	0.58	0.48–0.69	< 0.001
>78.2 ≤ 86.8	973 (22.9)	572 (22.4)	401	0.59	0.48–0.69	< 0.001
>86.8 ≤ 95.4	811 (19.1)	421 (16.5)	390	0.52	0.44–0.60	< 0.001
>95.4 ≤ 104	81 (1.9)	43 (1.7)	38	0.53	0.41–0.65	< 0.001

^1^Percentage of attendances in age band compared to total for all age bands

The mean Elixhauser co-morbidity score for non-COVID-19 medical admissions increased from 5.3 before the pandemic to 7.1 during the pandemic (*p* < 0.001) and within the pandemic first wave did not differ significantly from that of 6.8 for COVID-19 [25]. Patients admitted with non-COVID-19 diagnoses during the pandemic first wave had significantly higher levels of hypertension, heart failure, chronic kidney disease, cerebrovascular disease, liver disease and obesity compared to patients admitted before the pandemic (Table 3). During the first wave, COVID-19 admissions had significantly higher rates of diabetes, chronic pulmonary disease, obesity, and dementia, but lower rates of non-metastatic and metastatic cancer than non-COVID-19 admissions (Table 4).

**Table 3:** Number of admissions with a specific co-morbidity in patients admitted during the first wave with non-COVID-19 diagnoses and the matched pre-pandemic period

Characteristic	Pre-pandemic (%^1^)	1st pandemic wave (%^1^)	*P* value^2^
Dementia	537 (13)	283 (11)	0.052
Cerebrovascular disease	293 (6.9)	261 (10)	< 0.001
Myocardial infarction	408 (9.6)	286 (11)	0.038
Alcohol related liver disease	54 (1.3)	58 (2.3)	0.002
Hypertension	1782 (42)	1199 (47)	< 0.001
Diabetes	917 (22)	558 (22)	0.8
Chronic pulmonary disease	975 (23)	597 (23)	0.7
Heart failure	584 (14)	430 (17)	< 0.001
Liver disease	176 (4.1)	194 (7.6)	< 0.001
Kidney disease	594 (14)	400 (16)	0.063
Metastatic cancer	190 (4.5)	101 (4.0)	0.3
Non-metastatic solid organ tumour	302 (7.1)	150 (5.9)	0.045
Obesity	117 (2.8)	149 (5.8)	< 0.001

^1^ Refers to the percentage of all admissions during that time period

^2^ Pearson’s Chi-squared test

**Table 4:** Number of admissions with a specific co-morbidity in patients admitted during the pandemic first wave with or without a primary diagnosis of COVID-19

Characteristic	1st pandemic wave Non-COVID-19 (%)^1^	COVID-19 (%)^1^	*P* value^2^
Alcoholic liver disease	58 (2.3)	2 (0.4)	0.007
Dementia	283 (11)	87 (18)	< 0.001
Cerebrovascular disease	261 (10)	44 (9.0)	0.4
Myocardial infarction	286 (11)	39 (8.0)	0.036
Hypertension	1199 (47)	244 (50)	0.2
Diabetes	558 (22)	132 (27)	0.012
Chronic pulmonary disease	597 (23)	137 (28)	0.026
Heart failure	430 (17)	66 (14)	0.071
Liver disease	194 (7.6)	22 (4.5)	0.015
Kidney disease	400 (16)	82 (17)	0.5
Metastatic cancer	101 (4.0)	6 (1.2)	0.003
Non-metastatic solid tumour	150 (5.9)	18 (3.7)	0.053
Obesity	149 (5.8)	45 (9.2)	0.005

^1^ Refers to the percentage of all admissions during that time period

^2^Pearson’s Chi-squared test

### Physiological illness severity (NEWS2)

**Fig. 4:**
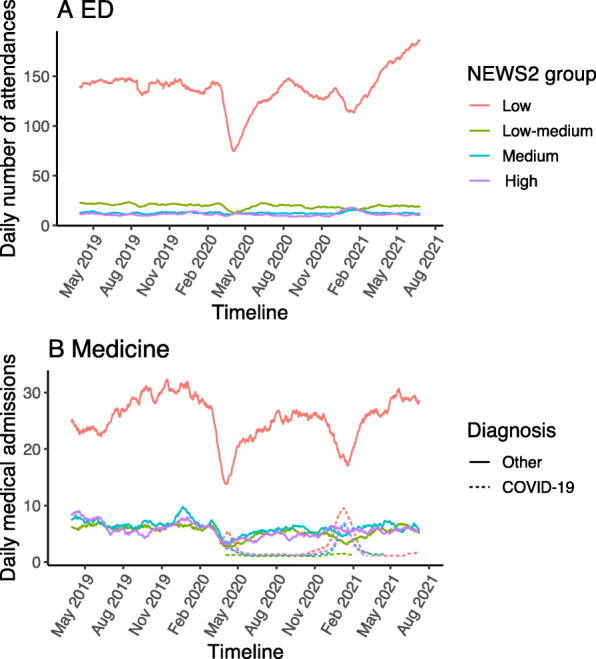
Physiological severity over time based on NEWS2 score risk alert level. (**A**) Total ED attendances. (**B**) Medical admissions stratified according to whether a primary diagnosis was COVID-19

Incident rates for ED attendances with low or low-medium NEWS2 severity score decreased significantly during the pandemic first waves, but there was no change in the incident rates for patients with medium or high severity scores (Table 5, Fig. 4A). Figure 4A demonstrates a small drop in low severity cases across the second wave with a rise in low severity cases in the summer of 2021. The overall mean severity score was 2.02 in the pre-pandemic period and 2.38 in the pandemic period for the whole of ED (*p* value < 0.001, Supplementary Table 3). A greater number of ED attendances required supplemental oxygen on presentation in the pandemic first wave period compared to the matched period in 2019 (4.4% vs 7.3%, *p* < 0.001, Supplementary Table 3).

**Table 5:** Number of ED attendees stratified by NEWS2 severity score group

NEWS2 Score	Pre-pandemic (%^1^)	1st pandemic wave (%^1^)	Difference	Incidence rate ratio	Confidence interval	*P* value
Low	10,841 (73.6)	7143 (69.0)	3698	0.66	0.59–0.73	< 0.001
Low-medium	2025 (13.8)	1465 (14.1)	560	0.72	0.64–0.81	< 0.001
Medium	980 (6.7)	898 (8.7)	82	0.92	0.81–1.02	0.14
High	875 (5.9)	849 (8.2)	26	0.97	0.87–1.07	0.55

^1^Percent refers to proportion of attendees in a given NEWS2 score group

The number of medical admissions fell significantly across all severity categories for non-COVID-19 diagnoses during the first wave of the pandemic compared to the matched period of the previous year (Fig. 4B, Table 6). Figure 4B shows a marked fall in low severity cases in the first and second waves of the pandemic, with severity trends rising thereafter to pre-pandemic levels. The mean severity score for medical admissions fell from 3.7 in the previous year to 3.18 for non-COVID-19 admission in the first wave (*p* < 0.001) but was increased at 4.49 for COVID-19 admissions in the first wave (*p* < 0.001, Supplementary Table 4/5). The proportion of medical admissions requiring supplemental oxygen at presentation during the pandemic was 52% among COVID-19 patients compared with 18% among those without COVID-19 (*p* < 0.001, Supplementary Table 4/5).

**Table 6:** Number of medical admissions stratified by NEWS2 severity score group

NEWS2 Score	Pre-pandemic (%^1^)	1st pandemic wave (%^1^)	Difference	Incidence rate ratio	Confidence interval	*P* value
Low	1834 (48.9)	1370 (59.9)	464	0.75	0.64–0.85	< 0.001
Low-medium	728 (19.4)	328 (14.3)	400	0.45	0.37–0.53	< 0.001
Medium	566 (15.1)	305 (13.3)	261	0.54	0.46–0.62	< 0.001
High	620 (16.5)	283 (12.4)	337	0.46	0.38–0.54	< 0.001

^1^Percent refers to proportion of attendees in a given NEWS2 score group

### Changes in presenting complaint and primary diagnoses

**Fig. 5:**
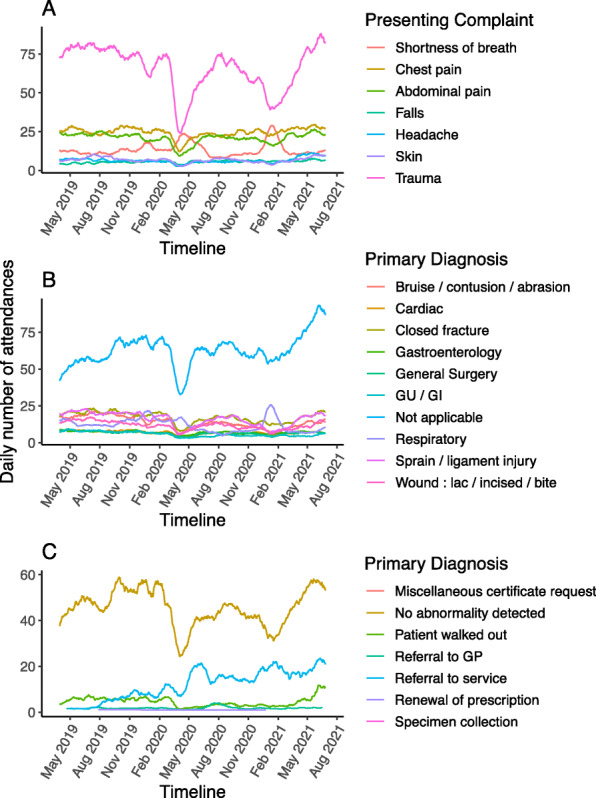
ED attendances by presenting complaint and diagnosis over time. (**A**) Daily attendances for the 10 commonest presenting complaints (‘Trauma’ includes injury of leg, arm, head injury and pain in the leg). (**B**) Daily attendances for the 10 commonest primary diagnoses. (**C**) Daily attendances for the group diagnosis of ‘not applicable’ broken down by subtype

During the first wave of the pandemic, the number of ED attendances with shortness of breath as the presenting complaint increased by 58% (Fig. 5A, Table 7). Of the 10 commonest presenting complaints, the others all decreased during the first wave except for falls (Table 7). There was a marked reduction in trauma in both the first and second COVID-19 waves (Fig. 5).

**Table 7:** Number of ED attendances in the pre and pandemic periods stratified by presenting complaint for the 10 most common presentations

Presenting complaint	Pre-pandemic	1st pandemic wave	Difference	Incidence rate ratio	Confidence interval	*P* value
Trauma – leg	2242	1034	1208	0.46	0.36–0.56	< 0.001
Trauma – arm	2044	1013	1031	0.50	0.38–0.61	< 0.001
Chest pain	2042	1301	741	0.64	0.56–0.72	< 0.001
Abdo pain	1790	934	856	0.52	0.45–0.59	< 0.001
Shortness of breath	960	1520	− 560	1.58	1.39–1.78	< 0.001
Head injury	921	501	420	0.54	0.46–0.63	< 0.001
Pain in leg	645	275	370	0.43	0.34–0.51	< 0.001
Headache	551	290	261	0.53	0.42–0.64	< 0.001
Skin	488	299	189	0.61	0.44–0.78	0.0004
Falls	333	355	−22	1.07	0.85–1.28	0.537

Among the 10 commonest groups of primary diagnosis in ED, only respiratory diagnoses (inclusive of COVID-19) did not fall significantly during the first wave (Table 8). Analysis of the most granular level of primary diagnosis demonstrated no reduction in the selected major diagnoses of cardiac arrests, pulmonary embolism, subarachnoid haemorrhage, acute renal failure, and diabetic ketoacidosis, but there were reductions in ST-elevation myocardial infarction, stroke, asthma, and vasovagal syncope (Table 9). There was a marked drop in the common diagnostic group of “not applicable” in the first wave followed by an overall increase in the summer 2021 (Fig. 5B). In most cases, this referred to a diagnosis of “no abnormality detected” or “patient walked out” (Fig. 5C). Attendances where the “patient walked out” fell to almost zero in the first wave and remained low until the summer of 2021 when they rose to the highest level (Fig. 5C).

**Table 8:** Number of ED attendances in the pre and pandemic periods stratified by primary diagnosis for the 10 most common primary diagnosis groups

Diagnosis	Pre-pandemic	1st pandemic wave	Difference	Incidence rate ratio	Confidence interval	*P* value
Not applicable	3760	3482	278	0.93	0.81–1.05	0.2446
Closed fracture	1522	808	714	0.53	0.44–0.62	<0.001
Sprain / ligament injury	1570	600	970	0.38	0.31–0.46	<0.001
Bruise / contusion / abrasion	1311	613	698	0.47	0.38–0.56	<0.001
Respiratory	1107	966	141	0.87	0.74–1.01	0.0852
Wound: lac / incised / bite	1119	582	537	0.52	0.46–0.58	<0.001
Gastroenterology	620	365	255	0.59	0.51–0.67	<0.001
Cardiac	634	347	287	0.55	0.5–0.6	<0.001
General Surgery	648	310	338	0.48	0.41–0.55	<0.001
GU / GI	620	243	377	0.39	0.33–0.45	<0.001

*lac* laceration, *GU / GI* genitourinary or gastrointestinal infection

**Table 9:** Number of ED attendances in the pre and pandemic period with selected specific ED diagnoses

Diagnosis	Pre-pandemic	1st pandemic wave	Difference	Incidence rate ratio	Confidence	*P* value
Cerebrovascular accident	181	121	60	0.67	0.48–0.86	0.006
Atrial fibrillation	170	122	48	0.72	0.5–0.93	0.028
Vasovagal syncope	165	78	87	0.47	0.3–0.65	< 0.001
Asthma	120	67	53	0.56	0.41–0.7	< 0.001
Acute coronary syndrome	89	41	48	0.46	0.32–0.6	< 0.001
Pulmonary embolism	85	61	24	0.72	0.44–0.99	0.087
Acute non-ST segment elevation myocardial infarction	50	37	13	0.74	0.35–1.13	0.259
Subarachnoid intracranial haemorrhage	30	24	6	0.80	0.46–1.14	0.299
Acute renal failure syndrome	29	23	6	0.79	0.49–1.09	0.226
Cardiac arrest	26	19	7	0.73	0.27–1.19	0.324
Acute ST segment elevation myocardial infarction	23	7	16	0.30	0.05–0.56	0.005
Ketoacidosis in diabetes mellitus	23	27	−4	1.17	0.5–1.85	0.58

**Fig. 6:**
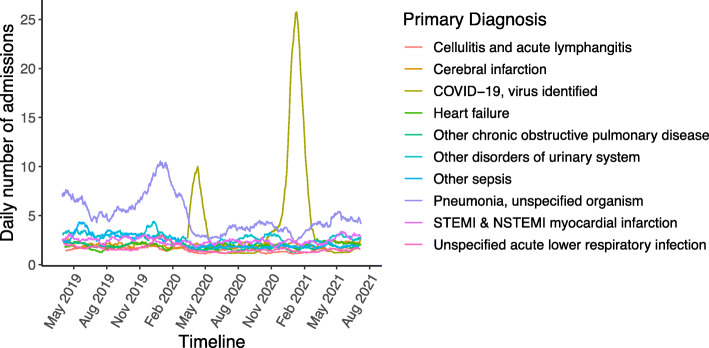
Daily medical admissions stratified by primary diagnosis for the 10 commonest diagnoses

For medical admissions, the most common primary diagnosis during the pandemic first wave period was ‘COVID-19, virus identified’, whereas at other times it was ‘pneumonia (unspecified organism)’ (Fig. 6, Table 10). The incidence of admissions for the other top 10 medical diagnoses, other than cerebral infarction, were significantly reduced during the first wave (Table 10). Cerebral infarction showed a trend to higher levels than the pre-pandemic period in the final months of the study period (Fig. 6, Supplementary Fig. 1). There was a marked peak in pneumonia in the winter of 2019–2020 which was not repeated the following winter when there was a second COVID-19 wave (Fig. 6).

**Table 10:** Incidence of top 10 most common primary diagnoses in admitted medical patients in the pre and pandemic first wave period

Diagnosis	Pre-pandemic	1st pandemic wave	Difference	Incidence rate ratio	Confidence	*P* value
Pneumonia, unspecified organism	518	204	314	0.39	0.31–0.48	< 0.001
Other disorders of urinary system	234	93	141	0.40	0.31–0.49	< 0.001
Myocardial infarction (with or without ST- elevation)	186	129	57	0.69	0.49–0.90	0.016
COVID-19, virus identified	NA	NA	NA	NA	NA	NA
Other sepsis	232	97	135	0.42	0.32–0.52	< 0.001
Cerebral infarction	129	124	5	0.96	0.69–1.23	0.782
Other chronic obstructive pulmonary disease	130	57	73	0.44	0.29–0.59	< 0.001
Heart failure	117	74	43	0.63	0.43–0.83	0.004
Unspecified acute lower respiratory infection	110	44	66	0.40	0.25–0.55	< 0.001
Cellulitis and acute lymphangitis	86	44	42	0.51	0.35–0.67	< 0.001

### Impact of the pandemic on outcomes

The mean length of stay for all ED attendances fell from 3.8 h to 3.1 h in the pandemic first wave period (*p* < 0.001, Student’s t test). For medical admissions, the mean length of stay was 6.2 days in the pre-pandemic period compared to 5.5 for non-COVID-19 admissions during the pandemic first wave (*p* < 0.001), and 9.6 days for COVID-19 admissions (*p* < 0.001 compared to non-COVID-19 first wave admissions).

**Fig. 7:**
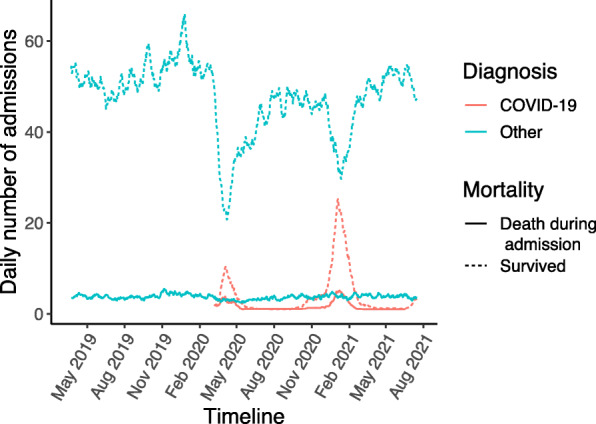
Daily medical admissions stratified by primary diagnosis and mortality

For ED attendances, the mortality rate during ED attendance or an associated admission was 1.2% (290/23218) in the pre-pandemic period compared to 2.9% (430/14398) in the pandemic first wave period (*p* < 0.001). Among medical admissions the overall inpatient mortality rate was 6.9% (292/4242) in the pre-pandemic period and 8.1% (207/2556) in the pandemic period among non-COVID-19 patients (*p* = 0.06). During the pandemic first wave, the inpatient mortality rate was higher for COVID-19 admissions at 28% (137/488, *p* < 0.001 compared to non-COVID-19 first wave admissions). Figure 7 demonstrates a relatively stable number of admissions of patients who died in hospital of non-COVID-19 illness, but a reduction in both COVID-19 waves in the number of patients admitted who did not die during the admission (Fig. 7).

## Discussion

Beyond the direct effect of COVID-19 illness, there have been concerns that the pandemic has deterred hospital attendances for acute non-COVID-19 illness, triggering increased downstream mortality or morbidity due to late presentation of disease [7, 8, 26–30]. In the UK, despite an attenuation in the pandemic from the spring of 2021, hospitals have experienced unprecedented ED attendances over the usually quiet summer period. In this context, understanding how the pandemic influenced hospital attendance and admission and which groups were under or over-represented is important for future health and pandemic planning.

As anticipated, ED attendances and medical admissions fell rapidly with the first UK death and the lockdown. In the UK, medical attendances are mostly referred by general practitioners or ED doctors and the rapid fall with the first wave suggests reduced referrals and/or a reduced acceptance rate. ED attendances and medical admissions fell less sharply in the second wave, despite higher numbers of COVID-19 cases and higher daily death rates, indicating acclimatisation to the pandemic by clinicians and patients.

ED attendances have a high proportion of young patients, whereas medical admissions have a high proportion of older patients [31, 32]. The fall in ED attendances by young patients during the pandemic reflects experience elsewhere and is likely multifactorial [26, 33]. In some areas, closure of higher education institutions will have prompted some students to return to their family homes [34, 35]. However, similar falls have been seen elsewhere and may be consistent with younger patients not attending ED with self-limiting conditions and possibly experiencing less minor trauma with reductions in some activities during the lockdown [7, 10]. The greatest relative reduction in medical admissions were in the oldest age groups possibly due to concerns about COVID-19 acquisition in hospital or about the futility of admission [36].

The increase in co-morbidities among medical admissions without COVID-19 suggests that admission for highly co-morbid patients was not easily avoidable, whilst those with lower co-morbidities presumably remained in the community. Medical admission with COVID-19 had a distinct comorbidity profile compared to non-COVID-19 admissions. Metastatic cancer was less common, perhaps because these patients shielded themselves from infections or because of reduced referral or admission rates; there is evidence of increased non-COVID-19 cancer mortality in the community [37]. In contrast, the increased prevalence of dementia among COVID-19 admissions could reflect outbreaks in nursing homes or difficulty shielding effectively when reliant on external carers. Other factors with higher prevalence in COVID-19 admissions, such as obesity and diabetes, reflect known susceptibility factors for severe COVID-19 illness [38, 39].

The NEWS2 severity scoring system has been adopted by the NHS to highlight patients at high risk of clinical deterioration [40–43]. The fall in lower severity ED attendances that we observed during the first wave is consistent with several other studies identifying the greatest reductions in low acuity attendances though these studies also identify some reductions in higher severity cases [44–46]. In a healthcare system in the Netherlands where there are generally fewer low acuity ED attendances, a 30% reduction in attendance was nevertheless still observed [28]. Reduced attendances among lower acuity ED patients may not be immediately harmful if they represent non-severe illness. However, the reductions we observed in medical admissions across all severity categories suggests that some patients with severe illness did not attend hospital.

Several factors might have contributed to the steady rise in low severity ED attendances after the second peak, including diminished concern about acquiring COVID-19 in hospital, especially in the vaccinated, publicity about the dangers of avoiding acute care, increased exercise and traffic trauma, symptoms of long COVID-19 and a perceived reduction in access to primary care which has experienced increased demand in additional to providing vaccines. Delayed presentations of major illness might be expected to result in greater numbers of patients with higher severity scores subsequently, but this was not observed. Nor was there an increase in cardiac arrest presentations, although this may have been influenced by increased attention to resuscitation status during the pandemic [47].

The marked reduction in ED attendances for trauma during the first wave likely reflects in part reduced physical activity, sport, and road traffic. UK road traffic dropped by 59% and a Spanish trauma unit described significant reduction in most trauma [48, 49]. The common ED diagnoses of ‘no abnormality detected’ dropped substantially during each COVID-19 peak, which is unlikely to have led to clinical harm and may suggest scope for reducing ED attendances in the future. During the first wave, there were almost no ED attendances where the patient walked out and walk-outs remained low for some time thereafter. This might reflect a faster ED service, as length of ED stay was reduced, or resulted from non-attendance of people with low severity complaints. Walkouts increased in the summer of 2021 in association with increased ED attendances.

There were no changes in ED attendances during the pandemic for certain conditions that might be acutely disabling, such as subarachnoid haemorrhage or diabetic ketoacidosis. Other centres observed either a reduction or no change in sub-arachnoid haemorrhage [50, 51]. In aggregate, there were reductions in cardiac diagnoses and at a granular level there were reductions in atrial fibrillation as well as myocardial infarction, with potential downstream consequences, for example if fewer people with atrial fibrillation received stroke preventing anticoagulants. We observed no change in cerebral infarction strokes during the first wave, although a study in Israel found a reduction of 29% for stroke and a UK-wide registry study found only a 12% reduction in stroke admissions at the peak of the first wave [52, 53]. Our data suggest a possible trend towards increased strokes in the last months of our analysis which, if confirmed over time, might reflect reduced opportunities for stroke prevention during the pandemic. Medical admissions for myocardial infarction, with and without ST-elevation, were reduced during the pandemic and this is consistent with reports from the UK and elsewhere [7, 9, 54–56]. The relatively constant inpatient mortality for non-COVID-19 medical admissions throughout the study might suggest that in our catchment area similar numbers of patients with the most severe life-threatening non-COVID-19 conditions were attending hospital throughout. However, nationally there is evidence of reductions in hospital mortality with concomitant increases in community mortality [37].

There are necessarily limitations to any retrospective study, and we cannot determine the precise factors that influenced whether a patient attended hospital and then was or was not admitted. ED diagnoses are provided by the initial clinician, often a junior clinician, and therefore may not be as robust as the diagnoses for medical admissions, which are ratified retrospectively by professional coders. The NEWS2 score had not yet been fully adopted by our institution during the first wave of COVID-19, so scores were not explicitly available to clinicians and were calculated retrospectively.

## Conclusions

The overall reduction in non-COVID-19 related acute presentations suggests both opportunities for reducing unnecessary ED attendances for those with low severity illness in the future, but also the possibility of harm from delayed or missed care. Trends to increased attendances and admissions during the recent summer 2021 period raise the possibility of further healthcare challenges, but also provide some reassurance that any deterrent effect on attendance is waning.

## Supplementary Information

**Additional file 1.** Supplementary Information - Additional tables for ED and medical admissions comprising absolute numbers with proportions for age, ethnicity, deprivation status, Oxygen usage and NEWS2 score. Supplementary figure 1 primary diagnosis for medical admissions split into individual panels for each diagnosis.

**Additional file 2.** Supplementary file ECDS data - Spreadsheet of emergency care dateset diagnoses and grouping structure.

## Data Availability

The raw data analysed during the current study are not publicly available to avoid any possibility of identification of local patients, but aggregated or limited data may be available from the corresponding author on reasonable request.

## Abbreviations

ED: emergency department
