# Supplementary material for: Impact of the COVID-19 pandemic on emergency department attendances and acute medical admissions
Source: BMC Emerg Med. 2021 Nov 20;21:143. doi: 10.1186/s12873-021-00529-w (PMC8605447; doi:10.1186/s12873-021-00529-w)
Supplement: Supplementary file 1 — Additional file 1. Supplementary Information - Additional tables for ED and medical admissions comprising absolute numbers with proportions for age, ethnicity, deprivation status, Oxygen usage and NEWS2 score. Supplementary figure 1 primary diagnosis for medical admissions split into individual panels for each diagnosis. [file 12873_2021_529_MOESM1_ESM.docx]

# Supplementary Information

## Supplementary Table 1 Counts of attendances to ED before and during the pandemic period stratified by age, ethnicity and deprivation decile. For ethnic groups a row-wise test of proportion p value is shown for individual categories in addition to the overall chi-squared test.

| Characteristic | Overall, N = 38,336^1^ | During 1^st^ wave, N = 14,828^1^ | Pre-pandemic, N = 23,508^1^ | p-value^2^ |
| --- | --- | --- | --- | --- |
| Age Group |  |  |  | <0.001 |
| [18,26.7] | 6,470 (17%) | 1,951 (13%) | 4,519 (19%) |  |
| (26.7,35.4] | 5,856 (15%) | 2,129 (14%) | 3,727 (16%) |  |
| (35.4,44.1] | 4,479 (12%) | 1,794 (12%) | 2,685 (11%) |  |
| (44.1,52.8] | 4,162 (11%) | 1,740 (12%) | 2,422 (10%) |  |
| (52.8,61.5] | 4,583 (12%) | 1,933 (13%) | 2,650 (11%) |  |
| (61.5,70.2] | 3,499 (9.1%) | 1,448 (9.8%) | 2,051 (8.7%) |  |
| (70.2,78.9] | 3,385 (8.8%) | 1,401 (9.4%) | 1,984 (8.4%) |  |
| (78.9,87.6] | 3,748 (9.8%) | 1,547 (10%) | 2,201 (9.4%) |  |
| (87.6,96.3] | 1,994 (5.2%) | 827 (5.6%) | 1,167 (5.0%) |  |
| (96.3,105] | 152 (0.4%) | 58 (0.4%) | 94 (0.4%) |  |
| Unknown | 8 | 0 | 8 |  |
| Mean age | 51 | 53 | 50 | <0.001 |
| Gender |  |  |  | 0.8 |
| Female | 19,607 (51%) | 7,594 (51%) | 12,013 (51%) |  |
| Male | 18,729 (49%) | 7,234 (49%) | 11,495 (49%) |  |
| Ethnic Group |  |  |  | <0.001 |
|  |  |  |  | Row-wise |
| Asian - Any Other Asian Background | 444 (1.2%) | 183 (1.2%) | 261 (1.1%) | 1 |
| Asian or Asian British - Bangladeshi | 90 (0.2%) | 38 (0.3%) | 52 (0.2%) | 1 |
| Asian or Asian British - Indian | 319 (0.8%) | 128 (0.9%) | 191 (0.8%) | 1 |
| Asian or Asian British - Pakistani | 423 (1.1%) | 143 (1.0%) | 280 (1.2%) | 0.6 |
| Black - Any Other Black Background | 175 (0.5%) | 73 (0.5%) | 102 (0.4%) | 1 |
| Black or Black British - African | 279 (0.7%) | 108 (0.7%) | 171 (0.7%) | 1 |
| Black or Black British - Caribbean | 115 (0.3%) | 35 (0.2%) | 80 (0.3%) | 0.9 |
| Mixed - Any Other Mixed Background | 196 (0.5%) | 86 (0.6%) | 110 (0.5%) | 1 |
| Mixed - White and Asian | 107 (0.3%) | 57 (0.4%) | 50 (0.2%) | 0.04 |
| Mixed - White and Black African | 71 (0.2%) | 31 (0.2%) | 40 (0.2%) | 1 |
| Mixed - White and Black Caribbean | 102 (0.3%) | 30 (0.2%) | 72 (0.3%) | 0.8 |
| Other - Any Other Ethnic Group | 393 (1.0%) | 162 (1.1%) | 231 (1.0%) | 1 |
| Other - Chinese | 142 (0.4%) | 38 (0.3%) | 104 (0.4%) | 0.07 |
| Other - Not Known | 371 (1.0%) | 121 (0.8%) | 250 (1.1%) | 0.258 |
| Other - Not Stated | 5,966 (16%) | 2,351 (16%) | 3,615 (15%) | 1 |
| White - Any Other White Background | 3,494 (9.1%) | 1,142 (7.7%) | 2,352 (10%) | <0.001 |
| White - British | 25,430 (66%) | 10,006 (67%) | 15,424 (66%) | 0.003 |
| White - Irish | 219 (0.6%) | 96 (0.6%) | 123 (0.5%) | 1 |
| Decile |  |  |  | 0.4 |
| 1 | 412 (1.1%) | 154 (1.1%) | 258 (1.1%) |  |
| 2 | 2,326 (6.2%) | 886 (6.1%) | 1,440 (6.3%) |  |
| 3 | 1,928 (5.1%) | 756 (5.2%) | 1,172 (5.1%) |  |
| 4 | 2,212 (5.9%) | 834 (5.7%) | 1,378 (6.0%) |  |
| 5 | 3,160 (8.4%) | 1,207 (8.3%) | 1,953 (8.5%) |  |
| 6 | 3,768 (10%) | 1,440 (9.8%) | 2,328 (10%) |  |
| 7 | 6,181 (16%) | 2,381 (16%) | 3,800 (17%) |  |
| 8 | 4,866 (13%) | 1,944 (13%) | 2,922 (13%) |  |
| 9 | 5,873 (16%) | 2,348 (16%) | 3,525 (15%) |  |
| 10 | 6,793 (18%) | 2,676 (18%) | 4,117 (18%) |  |
| Unknown | 817 | 202 | 615 |  |
| ^1^n (%) | | | | |
| ^2^Pearson's Chi-squared test; Wilcoxon rank sum test; Kruskal-Wallis rank sum test | | | | |

## Supplementary Table 2 Counts for medical admissions showing age, gender, ethnicity and deprivation for the pre-pandemic period and first wave of the pandemic stratified by COVID-19 and non-COVID-19 primary diagnosis.

| Characteristic | COVID-19, N = 488^1^ | During, N = 2,556^1^ | Pre, N = 4,242^1^ | p-value^2^ | Row-wise p value pre vs during (Non-COVID-19) | Row-wise p value non-COVID-19 vs COVID-19 during. |
| --- | --- | --- | --- | --- | --- | --- |
| Age Groups |  |  |  | <0.001 |  |  |
| [18,26.6] | 4 (0.8%) | 84 (3.3%) | 136 (3.2%) |  |  |  |
| (26.6,35.2] | 14 (2.9%) | 98 (3.8%) | 154 (3.6%) |  |  |  |
| (35.2,43.8] | 17 (3.5%) | 120 (4.7%) | 176 (4.1%) |  |  |  |
| (43.8,52.4] | 51 (10%) | 207 (8.1%) | 263 (6.2%) |  |  |  |
| (52.4,61] | 82 (17%) | 260 (10%) | 383 (9.0%) |  |  |  |
| (61,69.6] | 49 (10%) | 291 (11%) | 477 (11%) |  |  |  |
| (69.6,78.2] | 86 (18%) | 460 (18%) | 788 (19%) |  |  |  |
| (78.2,86.8] | 104 (21%) | 572 (22%) | 973 (23%) |  |  |  |
| (86.8,95.4] | 75 (15%) | 421 (16%) | 811 (19%) |  |  |  |
| (95.4,104] | 6 (1.2%) | 43 (1.7%) | 81 (1.9%) |  |  |  |
| Mean age | 69 | 69 | 71 | <0.001 |  |  |
| Gender |  |  |  | 0.7 |  |  |
| Female | 231 (48%) | 1,195 (47%) | 2,035 (48%) |  |  |  |
| Male | 251 (52%) | 1,336 (53%) | 2,178 (52%) |  |  |  |
| Unknown | 6 | 25 | 29 |  |  |  |
| Deprivation Decile |  |  |  | 0.4 |  |  |
| 1 | 3 (0.6%) | 21 (0.8%) | 42 (1.0%) |  |  |  |
| 2 | 31 (6.5%) | 156 (6.2%) | 211 (5.1%) |  |  |  |
| 3 | 23 (4.8%) | 91 (3.6%) | 156 (3.8%) |  |  |  |
| 4 | 17 (3.5%) | 136 (5.4%) | 205 (4.9%) |  |  |  |
| 5 | 28 (5.8%) | 195 (7.8%) | 327 (7.9%) |  |  |  |
| 6 | 53 (11%) | 239 (9.5%) | 396 (9.5%) |  |  |  |
| 7 | 88 (18%) | 398 (16%) | 714 (17%) |  |  |  |
| 8 | 64 (13%) | 336 (13%) | 541 (13%) |  |  |  |
| 9 | 88 (18%) | 441 (18%) | 706 (17%) |  |  |  |
| 10 | 85 (18%) | 497 (20%) | 856 (21%) |  |  |  |
| Unknown | 8 | 46 | 88 |  |  |  |
| Ethnic Group |  |  |  | <0.001 |  |  |
| Asian - Any Other Asian Background | 12 (2.5%) | 17 (0.7%) | 27 (0.6%) |  | 1 | 0.008 |
| Asian or Asian British - Bangladeshi | 2 (0.4%) | 9 (0.4%) | 5 (0.1%) |  | 1 | 1 |
| Asian or Asian British - Indian | 6 (1.2%) | 15 (0.6%) | 19 (0.5%) |  | 1 | 1 |
| Asian or Asian British - Pakistani | 3 (0.6%) | 21 (0.8%) | 31 (0.7%) |  | 1 | 1 |
| Black - Any Other Black Background | 1 (0.2%) | 4 (0.2%) | 12 (0.3%) |  | 1 | 1 |
| Black or Black British - African | 10 (2.1%) | 14 (0.6%) | 23 (0.5%) |  | 1 | 0.02 |
| Black or Black British - Caribbean | 2 (0.4%) | 6 (0.2%) | 9 (0.2%) |  | 1 | 1 |
| Mixed - Any Other Mixed Background | 3 (0.6%) | 6 (0.2%) | 14 (0.3%) |  | 1 | 1 |
| Mixed - White and Asian | 1 (0.2%) | 6 (0.2%) | 7 (0.2%) |  | 1 | 1 |
| Mixed - White and Black African | 2 (0.4%) | 0 (0%) | 7 (0.2%) |  | 1 | 0.3 |
| Mixed - White and Black Caribbean | 2 (0.4%) | 3 (0.1%) | 8 (0.2%) |  | 1 | 1 |
| Other - Any Other Ethnic Group | 6 (1.2%) | 11 (0.4%) | 19 (0.5%) |  | 1 | 0.8 |
| Other - Chinese | 2 (0.4%) | 2 (<0.1%) | 7 (0.2%) |  | 1 | 1 |
| Other - Not Known | 3 (0.6%) | 13 (0.5%) | 17 (0.4%) |  | 1 | 1 |
| Other - Not Stated | 97 (20%) | 347 (14%) | 446 (11%) |  | 0.002 | 0.006 |
| White - Any Other White Background | 9 (1.9%) | 88 (3.5%) | 176 (4.2%) |  | 1 | 1 |
| White - British | 318 (66%) | 1,959 (77%) | 3,354 (80%) |  | 0.58 | <0.001 |
| White - Irish | 3 (0.6%) | 10 (0.4%) | 32 (0.8%) |  | 1 | 1 |
| Unknown | 6 | 25 | 29 |  |  |  |
| ^1^n (%); Mean, Median | | | | |  |  |
| ^2^Pearson's Chi-squared test; Kruskal-Wallis rank sum test | | | | |  |  |

## Supplementary Table 3 Oxygen usage and mean NEWS2 score with missing data counts for ED attendances stratified by pre or during the pandemic first wave.

| Characteristic | During, N = 14,828^1^ | Pre-pandemic, N = 23,508^1^ | p-value^2^ |
| --- | --- | --- | --- |
| Oxygen use |  |  | <0.001 |
| No | 10,234 (93%) | 15,183 (96%) |  |
| Yes | 804 (7.3%) | 692 (4.4%) |  |
| Unknown | 3,790 | 7,633 |  |
| Mean NEWS2 score | 2.38 | 2.02 | <0.001 |
| Unknown | 4,759 | 9,124 |  |
| ^1^n (%); Mean | | | |
| ^2^Pearson's Chi-squared test; Wilcoxon rank sum test | | | |

## Supplementary Table 4 Oxygen usage and NEWS2 score for medical admissions pre and during the pandemic first wave excluding COVID-19 admissions.

| Characteristic | During, N = 2,556^1^ | Pre-pandemic, N = 4,242^1^ | p-value^2^ |
| --- | --- | --- | --- |
| Oxygen use |  |  | 0.030 |
| No | 2,039 (82%) | 3,529 (84%) |  |
| Yes | 437 (18%) | 653 (16%) |  |
| Unknown | 80 | 60 |  |
| Mean NEWS2 score | 3.2 | 3.7 | <0.001 |
| Unknown | 373 | 741 |  |
| ^1^n (%); Mean | | | |
| ^2^Pearson's Chi-squared test; Wilcoxon rank sum test | | | |

## Supplementary Table 5 NEWS2 score and oxygen usage during the pandemic first wave comparing admissions with and without a primary diagnosis of COVID-19.

| Characteristic | COVID-19, N = 488^1^ | During, N = 2,556^1^ | p-value^2^ |
| --- | --- | --- | --- |
| Oxygen usage |  |  | <0.001 |
| No | 229 (48%) | 2,039 (82%) |  |
| Yes | 250 (52%) | 437 (18%) |  |
| Unknown | 9 | 80 |  |
| Mean NEWS2 score | 4.49 | 3.18 | <0.001 |
| Unknown | 38 | 373 |  |
| ^1^n (%); Mean | | | |
| ^2^Pearson's Chi-squared test; Wilcoxon rank sum test | | | |

Supplementary Figures


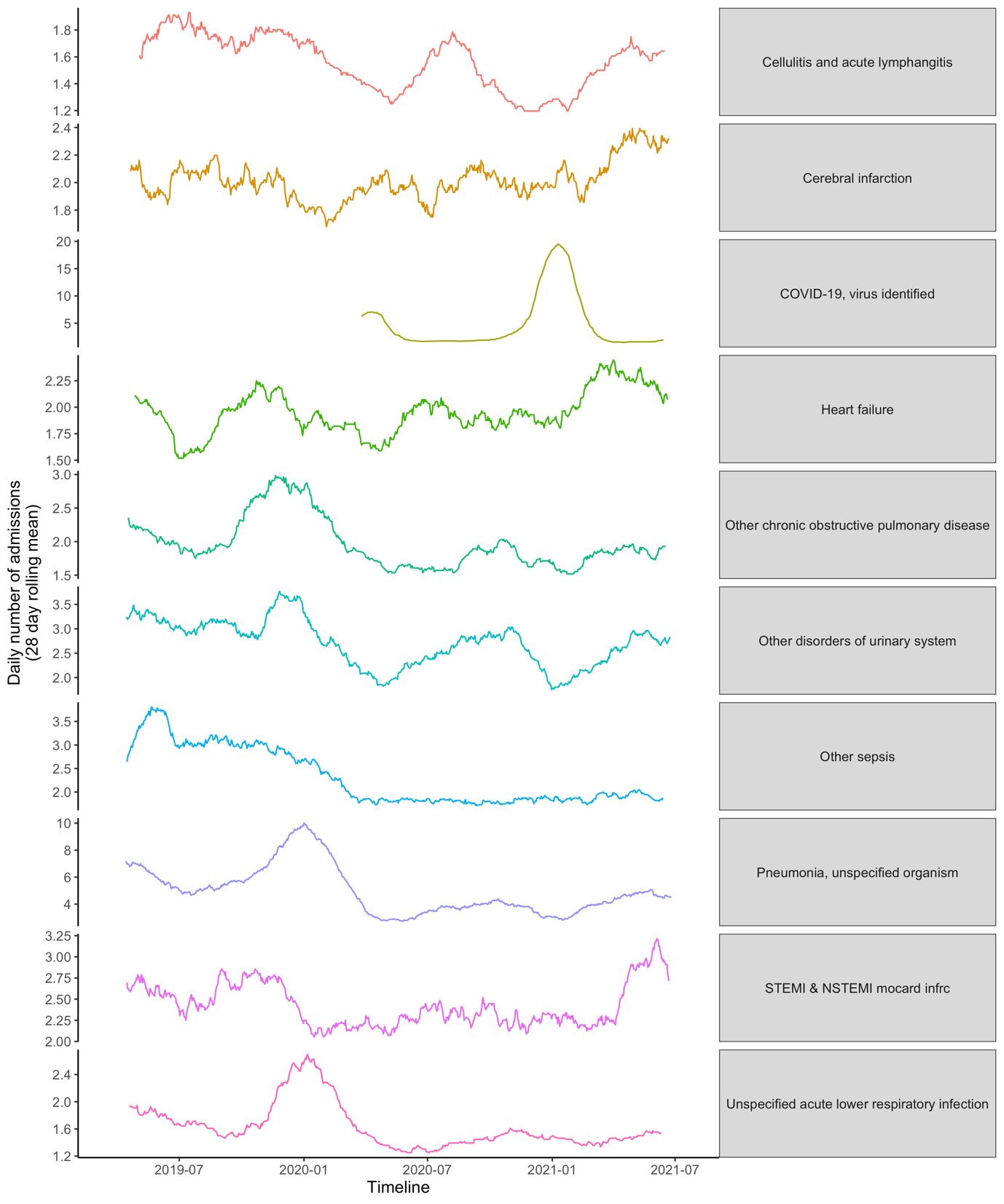


Supplementary Figure 1 Daily number of admissions to acute medicine stratified by primary diagnosis - data as per Figure 6 but shown in individual panels to aid inspection of individual diagnoses.
